# Simulation of the Transmission Spectrum of Long-Period Fiber Gratings Structures with a Propagating Acoustic Shock Front

**DOI:** 10.3390/s21217212

**Published:** 2021-10-29

**Authors:** Oleg V. Ivanov, Paulo Caldas, Gaspar Rego

**Affiliations:** 1 Ulyanovsk Branch of Kotel’nikov Institute of Radio Engineering and Electronics of Russian Academy of Sciences, Ulitsa Goncharova 48, 432071 Ulyanovsk, Russia; 2 Ulyanovsk State University, S.P. Kapitsa Research Institute of Technology, Ulitsa L. Tolstogo 42, 432017 Ulyanovsk, Russia; 3 ProMetheus-Research Unit in Materials, Energy and Environment for Sustainability, Instituto Politécnico de Viana do Castelo, 4900-347 Viana do Castelo, Portugal; pcaldas@estg.ipvc.pt (P.C.); gaspar@estg.ipvc.pt (G.R.); 4 Center for Applied Photonics, Institute for Systems and Computer Engineering, Technology and Science (INESC TEC), Rua Dr. Roberto Frias, 4200-465 Porto, Portugal

**Keywords:** long-period fiber grating, optical spectrum, fiber optics sensor, shock wave, shock front

## Abstract

In this paper, we investigate modification of transmission spectra of long-period fiber grating structures with an acoustic shock front propagating along the fiber. We simulate transmission through inhomogeneous long-period fiber gratings, π-shift and reflective π-shift gratings deformed by an acoustic shock front. Coupled mode equations describing interaction of co-propagating modes in a long-period fiber grating structures with inhomogeneous deformation are used for the simulation. Two types of apodization are considered for the grating modulation amplitude, such as uniform and raised-cosine. We demonstrate how the transmission spectrum is produced by interference between the core and cladding modes coupled at several parts of the gratings having different periods. For the π-shift long-period fiber grating having split spectral notch, the gap between the two dips becomes several times wider in the grating with the acoustic wave front than the gap in the unstrained grating. The behavior of reflective long-period fiber gratings depends on the magnitude of the phase shift near the reflective surface: an additional dip is formed in the 0-shift grating and the short-wavelength dip disappears in the π-shift grating.

## 1. Introduction

In the past two to three decades, fiber gratings have been intensively studied for applications in optical communications and sensing [1,2,3]. In the later domain, fiber Bragg gratings have also been used for detection of acoustic and ultrasound waves [4,5,6]. More recently, the scientific community focused their attention on the development of sensors able to detect deformations caused by shock waves working at frequencies from hundred kHz up to several GHz; examples of such acoustic shock waves are explosive detonation waves. In this context, fiber Bragg gratings have already proved their potential and several papers have been published in literature [7,8,9,10,11], while the interaction of a shock wave front with a fiber Bragg grating was also studied [12]. It would be of interest to investigate if similar methods could be used for long-period fiber gratings (LPFGs) and to analyze the difference between acoustic effects in short and long-period gratings. However, the use LPFGs for ultrafast applications is discussed only in scarce works related to the detection of ultrasound waves [13,14,15]. In general, the mechanism of detection relies on the effect of acoustic pressure on the grating that can be further amplified if the LPFG is bent. In fact, different configurations, included interferometric ones, can be employed for detection. So far, no theoretical work has been published regarding the effect of shock waves on LPFG transmission spectra.

Conversely, the interaction of ultrasound with grating sensors can be used to identify leakage of medical gas delivery systems in health care facilities. This issue has a tremendous financial impact, which is even more demanding in the current pandemic situation.

For the case of step-changed acoustic fronts, calculating transmission spectra is similar to the problem of inhomogeneous step-changed LPFGs where some part of the grating is strained, while the strain sensitivity of LPFGs has been analyzed in Ref. [16]; meanwhile, the properties of inhomogeneous LPFGs with apodization, chirp, and phase shift have been investigated in Refs. [17,18,19,20,21], and the step can be produced by concatenating two LPFGs with different periods [22,23], modulation amplitudes [24,25], and some phase shift between them [26]. Inhomogeneous gratings can be formed by heating part of the fiber grating and are used as gain-flattening filters [27].

The spectral response of LPFGs can be controlled by adding a phase shift in the middle of the grating [19,28,29], while the phase shift is formed by a section of fiber without grating, mechanically, or by the changes in its refractive index [30,31]. In contrast to homogeneous LPFGs, a pass band is created in the resonance notches of the phase-shifted LPFGs as a result of interference between two parts of the grating. The phase-shifted LPFGs have been used for sensing, in particular, for the measurements of temperature and refractive index [32], twist [33], strain [34], bending [23], as gain-flattening filters in erbium-doped fiber amplifiers [35,36], and optical differentiators [37]. Unique response characteristics of π-phase-shifted fiber Bragg gratings for ultrasonic detection have been described [38]. Higher sensitivity of the phase-shifted LPFG may be also advantageous when it is employed for the detection of acoustics waves.

The standard LPFG works in transmission and requires access to both end-sides of the fiber: one brings the light from a source and the other returns the remaining light to a photo-detector. In terms of sensing, it is more convenient to create a sensing head having access to a single end of the fiber. This can be attained by using a reflective surface at the end of the optical fiber with the LPFG in a self-interference Michelson configuration [39,40,41,42,43,44,45,46], and the size of the sensing head is halved in this case. An arbitrary phase shift can be introduced in the center of the grating, if a gap is added between the LPFG and the mirror. We can also expect different behavior of such a structure under the influence of an acoustic wave front.

In this work, we investigate modification of transmission spectra of long-period fiber grating structures with an acoustic shock front propagating along the fiber. We simulate transmission through homogeneous long-period fiber gratings, π-shift, and reflective gratings with propagating acoustic shock front producing non-uniform deformation along the optical fiber. Coupled mode equations describing interaction of co-propagating modes in a long-period fiber grating structures with inhomogeneous deformation are written and used for the simulation. We consider the influence of smoothness of the acoustic front and the magnitude of the phase shift on the spectra of various types of LPFG structures.

## 2. Long-Period Fiber Grating Structures

**Figure 1:**
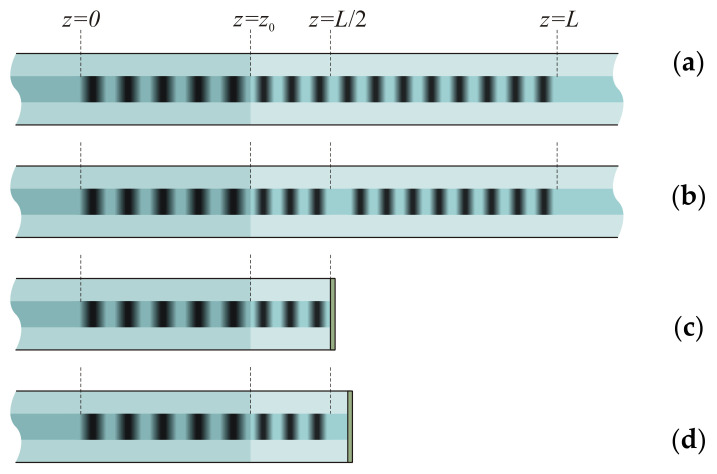
Scheme of LPFG structures deformed by an acoustic shock front: (**a**) homogeneous, (**b**) π-sift, (**c**) reflective, (**d**) reflective π-shift.

The long-period fiber grating has periodical modulation of its refractive index with period between 100 and 1000 μm (Figure 1a). The transmission spectrum of a LPFG is a function with several dips centered at resonance wavelengths, which are determined by the grating period and propagation constants of the core and cladding modes. The homogeneous LPFGs have symmetric dips with some sidelobes.

The phase-shifted LPFG has a section with modified fiber structure, where the grating has an additional space between the grating lines or changed propagation constants of the core and cladding modes (Figure 1b). Each spectral notch of the phase-shifted LPFG is split into two dips. The influence of the phase shift on the spectrum is strongest when it is in the center of grating. The relation between the amplitudes of the dips is determined by the magnitude of the phase shift. The amplitudes are equal for π phase shift; here, we consider only π-phase-shifted LPFGs in the center of the grating.

The reflective LPFG has a reflective coating at the end face of the optical fiber near the grating. If the grating ends exactly at the end face, no phase shift is introduced for the propagating modes (Figure 1c). Such a grating works as a single homogeneous LPFG with doubled length. However, its behavior deformed by acoustic wave differs from the homogeneous LPFG, because the acoustic wave covers the unwrapped grating starting from its center, not from one side.

If there is some space between the grating and the end face, a phase shift is formed (Figure 1d). We obtain a grating that is equivalent in unwrapped form to a phase-shifted grating or a cascaded grating, and again, when an acoustic wave propagates through the grating, it starts from the center of the unwrapped grating.

We assume that a longitudinal compressive shock front of acoustic shock wave propagates along the fiber and deforms the grating starting from z=L (Figure 1). The speed of the acoustic shock wave is defined by the speed of sound in the silica fiber. When the shock front propagates along the fiber with the LPFG, the grating is divided into two parts; one part is deformed and its period is decreased and the refractive index is changed due to strain-optic effect, while, in a homogeneous grating, this would shift the resonance wavelengths of the spectral peaks. The shift can be positive or negative for different cladding modes depending on specific fiber structure parameters [47,48]. The other part is unaffected and its period is not changed.

The front of the shock wave may have different profiles, which is determined by the time profile of the initial impact and by diffusion of the shock front due to dispersion of the speed of sound in the fiber and surrounding material. Assuming that the initial shock front is described by the step function, it can be represented as a Fourier transform of waves with different frequencies. These waves propagating in a dispersive medium have different speeds and arrive to the grating in different moments. The resulting sum of the waves is some smoothed stepwise function. The magnitude of smoothing is determined by the dispersion of the medium and the distance from the source to the grating. We consider only the effect of the leading edge of compression of the shock wave, which is much sharper compared to the trailing edge of expansion. Slow expansion has space distribution larger than the grating length and covers the whole grating, which acts as a homogeneous structure in this case.

The strain can be strong enough so that the wavelength shift is large and resonances of cladding mode with adjacent mode numbers can overlap. This is an alternative kind of interference, because cladding modes with different mode numbers do not interfere themselves; indeed, it would take place only interference between them when they couple back to the core mode. Another situation is possible when the LPFG excites modes of different types [49]: the overlap of resonances of different types of modes. We will not consider this situation in this paper.

The refractive index modulation in a fiber with a LPFG can be written as follows:

(1) $$n(r,z)=n_{0}(r)\left\{1+g(r)\sigma (z)\left[1+\cos({2\pi z/\Lambda }_{0})\right]\right\}$$

where $n_{0}(r)$ is the refractive index of the fiber without the grating, σ(z) is a slowly varying envelope of modulation amplitude of the induced refractive index, g(r) is the function describing radial dependence refractive index modulation (in photoinduced gratings, $g(r)=g_{\mathrm{co}}$ for $r\leq r_{\mathrm{co}}$ and g(r)=0 for $r>r_{\mathrm{co}}$), and $\Lambda_{0}$ is the grating period.

To simulate the process of propagation of acoustic shock front through the LPFG, we assume that the amplitude of the front profile is described by the following function, which changes the strain from one level to another with controllable smoothness defined by the parameter η:

(2) $$s(z)=\frac{s_{0}}{2}\left[1+\tanh\frac{z-z_{0}}{\eta }\right]$$

**Figure 2:**
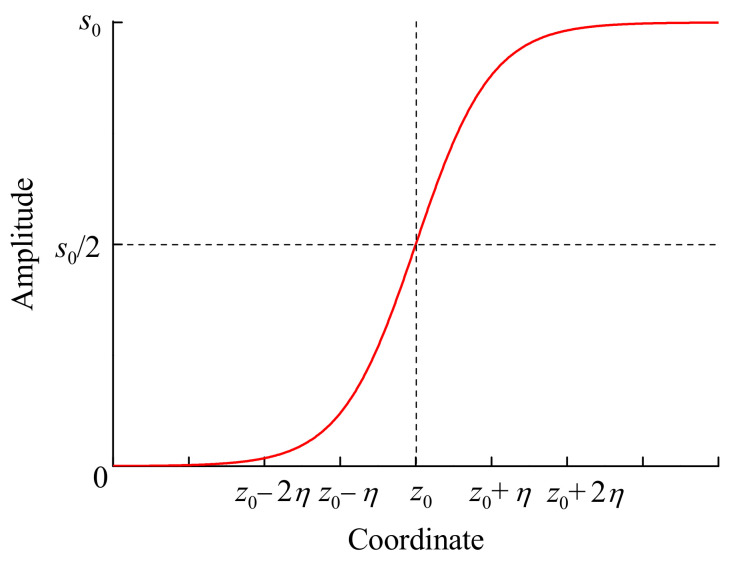
Longitudinal coordinate dependence of amplitude of the shock front.

Here, $z_{0}$ is the position of the shock front. Figure 2 illustrates the dependence of the amplitude of the shock front on the longitudinal coordinate. The shock front compresses the fiber, and each point of the fiber is displaced by u(z) in −z direction:

(3) $$u(z)=\int_{-\infty }^{z}s(z)\;dz$$

The refractive index modulation in the deformed fiber takes the form

(4) $$n(r,z)=n_{0}(r)\left\{1+g(r)\sigma \left(z+u(z)\right)\left[1+\cos\left({2\pi (z+u(z))/\Lambda }_{0}\right)\right]\right\}$$

The period of the deformed grating is determined by the function s(z)and decreases a result of strain: $\Lambda (z)=\Lambda_{0}(1-s(z))$.

In addition to displacement of the fiber grating, the refractive index of the fiber is modified due to strain-optic effect by

(5) $$\Delta n_{s}(r,z)=n_{0}(r)p_{a}(r)s(z)$$

where $p_{a}$ is the effective strain-optic coefficient $p_{a}=\frac{n^{2}}{2}\left(p_{12}-\nu (p_{11}-p_{12})\right)$, $p_{12}$ and $p_{11}$ are the strain-optic coefficients, and ν is the Poisson’s ratio. In contrast to fiber Bragg gratings, the dependence of the refractive index and the strain-optic coefficient in the right part of the Equation (5) on radius cannot be neglected. Otherwise, the core and cladding modes would have identical sensitivity to strain, and their contributions in the wavelength shift, as we show below, would cancel each other.

The dielectric permittivity and its change in the deformed and strained fiber can be expressed from Equation (4) as

(6) $$\begin{array}{l} \varepsilon (r,z)=n_{0}{\left(r\right)}^{2}\left(1+2p_{a}s(z)\right)\left\{1+2g(r)\sigma (z)\left[1+\cos\left({2\pi (z+u(z))/\Lambda }_{0}\right)\right]\right\}\\ \Delta \varepsilon (r,z)=2n_{0}{\left(r\right)}^{2}\left\{p_{a}s(z)+g(r)\sigma (z)\left[1+\cos\left({2\pi (z+u(z))/\Lambda }_{0}\right)\right]\right\} \end{array}$$

Here we have neglected the terms of the second order of smallness in the change of dielectric permittivity.

## 3. Coupled Mode Equations

The long-period fiber gratings couple modes propagating in the same direction. We consider the case of one core mode that is coupled to the forward propagating cladding modes. The interaction with backward propagating modes may occur only at the endface of the fiber for reflective LPFGs. The coupling coefficient between two co-propagating modes is defined as the following overlap integral over the fiber cross-section:

(7) $$K_{\mu \nu }(z)=\frac{\omega \varepsilon_{0}}{4}\int_{\infty }E_{\mu }^{*}(r,\varphi )\Delta \varepsilon (r,z)E_{\nu }(r,\varphi )dS$$

where Δε(r,z) is the perturbation of dielectric permittivity in the fiber as a result of grating inscription and propagating acoustic wave, $E_{\mu }(r,\varphi )$ and $E_{\nu }(r,\varphi )$ are the transverse distributions of electric field of HE_1__*μ*_ and HE_1*ν*_ modes, respectively, ω is the frequency, and $\varepsilon_{0}$ is the dielectric constant of vacuum.

The coupled mode equations that describe the amplitudes of modes can be written as

(8) $$\frac{dA_{\mu }}{dz}=\;i\sum_{\nu }^{}K_{\mu \nu }A_{\nu }\exp\left(i(\beta_{\nu }-\beta_{\mu })z\right)$$

where $\beta_{\mu }$ and $\beta_{\mu }$ are the propagation constants of the μ-th and ν-th modes. We represent the coupling coefficient, taking into account that the perturbation of dielectric permittivity depends on coordinate z, as

(9) $$K_{\mu \nu }(z)=\gamma_{\mu \nu }(z)+\kappa_{\mu \nu }(z)\left[1+\cos\left({2\pi (z+u(z))/\Lambda }_{0}\right)\right]$$

The two coupling constants $\gamma_{\mu \nu }(z)$ and $\kappa_{\mu \nu }(z)$ describe mode coupling due to strain and modulation in the grating, respectively:

(10) $$\gamma_{\mu \nu }(z)=\frac{\omega \varepsilon_{0}}{2}p_{a}s(z)\int_{0}^{r_{\mathrm{cl}}}n_{0}{\left(r\right)}^{2}E_{\mu }^{*}(r)E_{\nu }(r)2\pi rdr$$

(11) $$\kappa_{\mu \nu }(z)=\frac{\omega \varepsilon_{0}}{2}\sigma (z)\int_{0}^{r_{\mathrm{cl}}}n_{0}{\left(r\right)}^{2}g(r)E_{\mu }^{*}(r)E_{\nu }(r)2\pi rdr$$

We have assumed here that only HE_1_*_μ_* modes without azimuthal structure interact, because the fiber is straight and has no birefringence and the grating is radially symmetric. The coupled mode equations are obtained by ignoring quickly oscillating terms and describe slowly varying amplitudes of two co-propagation modes:

(12) $$\begin{array}{l} \frac{dA}{dz}=\;i\left[\gamma_{\mathrm{co}-\mathrm{co}}(z)+\kappa_{\mathrm{co}-\mathrm{co}}(z)\right]A+\frac{i}{2}\;\kappa_{\mathrm{co}-\nu }(z)B\exp(-i2\delta z)\\ \frac{dB}{dz}=\;i\left[\gamma_{\nu -\nu }(z)+\kappa_{\nu -\nu }(z)\right]B+\frac{i}{2}\;\kappa_{\mathrm{co}-\nu }(z)A\exp(i2\delta z) \end{array}$$

where A and B denote the amplitudes of co-propagating modes HE_11_ and HE_1_*_ν_*, respectively, and the detuning parameter is

(13) $$\delta (z)=\frac{1}{2}\left(\beta_{\mathrm{co}}-\beta_{\nu }\right)-\left(1+\frac{u(z)}{z}\right)\frac{\pi }{\Lambda_{0}}$$

For obtaining Equation (12), we use the equality of coupling constants $\kappa_{\mathrm{co}-\nu }=\kappa_{\nu -\mathrm{co}}$.

The resonance condition for mode coupling is

(14) $$\delta +\left(\gamma_{\mathrm{co}-\mathrm{co}}-\gamma_{\nu -\nu }+\kappa_{\mathrm{co}-\mathrm{co}}-\kappa_{\nu -\nu }\right)/2=0$$

The effective refractive indices $n_{}^{\left(\mathrm{eff}\right)}=\beta /k_{0}$ depend on wavelength ($k_{0}=\omega /c$ is the wavenumber in vacuum) and can be approximately written in a range of tens of nanometer around one of the cladding mode dips as $n^{\left(\mathrm{eff}\right)}(\lambda_{0})+\Delta \lambda dn^{\left(\mathrm{eff}\right)}/d\lambda$. Then, we write for the detuning parameter

(15) $$\delta =\frac{\pi \Delta \lambda }{\lambda_{0}^{}}\left[-\frac{n_{\mathrm{co}}^{\left(\mathrm{eff}\right)}-n_{\nu }^{\left(\mathrm{eff}\right)}}{\lambda_{0}}+\left(\frac{dn_{\mathrm{co}}^{\left(\mathrm{eff}\right)}}{d\lambda }-\frac{dn_{\nu }^{\left(\mathrm{eff}\right)}}{d\lambda }\right)\right]-\frac{u(z)}{z}$$

For simulation, we use parameters of SMF-28 fiber at wavelength $\lambda_{0}=1550$ nm: core radius $r_{\mathrm{co}}=4.2$ μm, cladding refractive index $n_{\mathrm{cl}}^{}=1.4440$, core refractive index $n_{\mathrm{co}}=1.4494$, and the effective refractive index of the core mode $n_{\mathrm{co}}^{\left(\mathrm{eff}\right)}=1.4465$. Integrating over the fiber cross-section, we obtain the following approximations for the coupling constants:

(16) $$\begin{array}{l} \gamma_{\mathrm{co}-\mathrm{co}}(z)=\left(1+\alpha_{\mathrm{co}-\mathrm{co}}\right)n_{\mathrm{cl}}^{}p_{a}s(z)k_{0}\\ \gamma_{\nu -\nu }(z)=\left(1+\alpha_{\nu -\nu }\right)n_{\mathrm{cl}}^{}p_{a}s(z)k_{0}\\ \kappa_{\mathrm{co}-\mathrm{co}}(z)=0.78\;n_{\mathrm{cl}}^{}\sigma (z)k_{0}\;\\ \kappa_{\nu -\nu }(z)=0\\ \kappa_{\mathrm{co}-\nu }(z)=\chi_{\nu }\;n_{\mathrm{cl}}^{}\sigma (z)k_{0} \end{array}$$

The coefficient 0.78 in the self-coupling constant $\kappa_{\mathrm{co}-\mathrm{co}}$ is obtained as the self-overlap intergal of the core mode over the fiber core. The overlap integrals $\gamma_{\mathrm{co}-\mathrm{co}}$ and $\gamma_{\nu -\nu }$ are equal to $n_{\mathrm{cl}}^{}p_{a}s(z)k_{0}$ with some small corrections $\alpha_{\mathrm{co}-\mathrm{co}}$ and $\alpha_{\nu -\nu }$, which are caused by several effects induced by deformation and strain in the fiber [12]. The cross-coupling coefficient $\kappa_{\mathrm{co}-\nu }$ depends on mode number via $\chi_{\nu }$: $\chi_{\nu }=0.030$, 0.055, 0.076, 0.092 for ν=2,3,4,5, respectively (see Appendix A).

As follows from Equation (14), when the fiber is strained, the wavelength shift of a spectral dip is determined by the difference of self-coupling constants depending on strain:

(17) $$\gamma_{\mathrm{co}-\mathrm{co}}-\gamma_{\nu -\nu }=\left(\alpha_{\mathrm{co}-\mathrm{co}}-\alpha {\;}_{\nu -\nu }\right)n_{\mathrm{cl}}^{}p_{a}s(z)k_{0}$$

We take $\alpha_{\mathrm{co}-\mathrm{co}}-\alpha {\;}_{\nu -\nu }=-0.01$, which is close to the first and largest overlap integral for the compression of the core refractive index profile under the deformation (see Equations 21 and 35 in [12]). As compared to fiber Bragg gratings, the effect of changing phase of the strained grating is smaller, because the period is smaller by several orders of magnitude. The mechanical displacement of the long-period grating end under strain is of the same order as the grating period.

## 4. Simulation of Uniform LPFG

We begin simulation with a uniform grating having constant modulation amplitude of induced refractive index along the length of the fiber. Let the grating has a period of 430 μm. Then the resonance of the fifth mode HE_15_ is centered at 1550 nm. At this wavelength, we can find the effective refractive index of the fifth cladding mode $n_{\mathrm{co}}^{\left(\mathrm{eff}\right)}=1.44306$. We choose such amplitude of refractive index modulation $\sigma =1.48\cdot 10^{-4}$ that the transmission in the center of resonance is equal to zero. This corresponds to the coupling constant between the core the fifth cladding mode $\kappa_{\mathrm{co}-5}=$$1.6\cdot 10^{-4}$ μm^–1^. All parameters that we used in the simulation are listed in Table 1. The numerical simulation was made in Matlab (R2012b).

**Figure 3:**
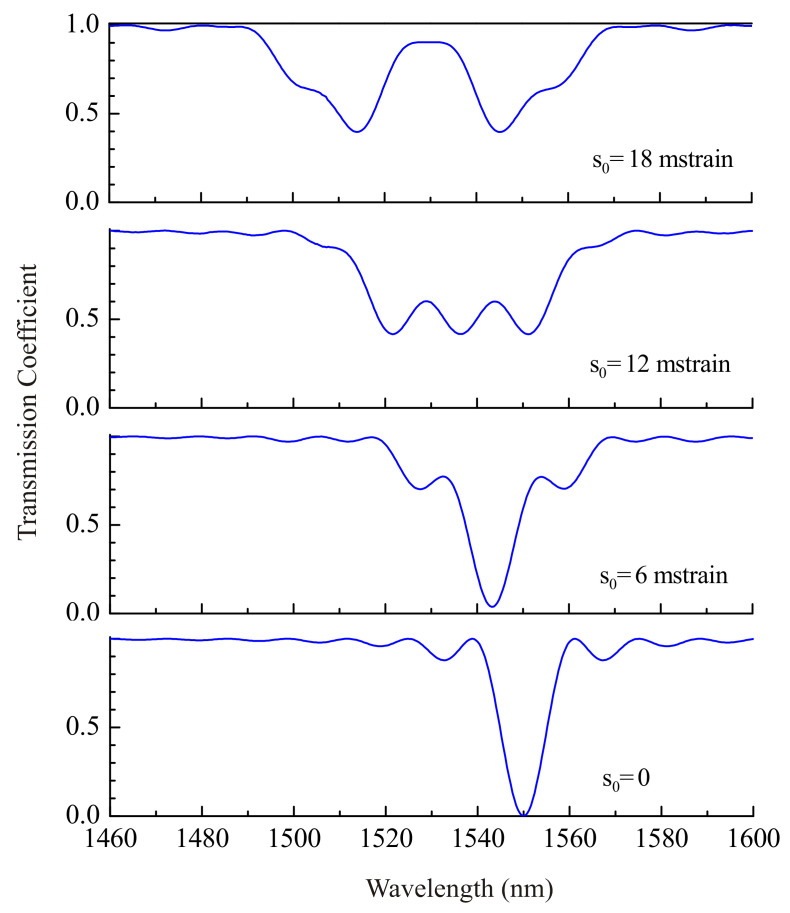
Transmission coefficient of grating for LP_05_ cladding mode, when the acoustic front is in its center $z_{0}=L/2$, for different strains $s_{0}=0$, 6, 12, and 18 mstrain.

The transmission spectra of a regular LPFG are shown in Figure 3 for different values of strain in stepwise acoustic front (η=0) located in the center of the grating $\left(z_{0}=L/2\right)$. The spectrum of the uniform grating without strain is shown in the lower plot. This spectral shape is a dip with minimum transmission in the center and represents the standard LPFG notch.

The acoustic front propagating through the grating divides the grating into two parts with different parameters. Such a grating is equivalent to a step-changed grating [22], and these parts have different resonance wavelengths. The core and cladding modes coupled at the two sections interfere and form an interference spectrum in transmission. It is symmetric, because the step is in the center of the grating. The spectrum shifts to shorter wavelengths with an increasing amplitude of the acoustic front due to decreasing average period of the grating. We can also see that the presence of the shock front in the fiber leads, first, to formation of a broader notch and, second, to splitting of the dip. For high strains, the spectrum completely splits into two dips. Each dip corresponds to its own homogeneous part of the grating.

**Figure 4:**
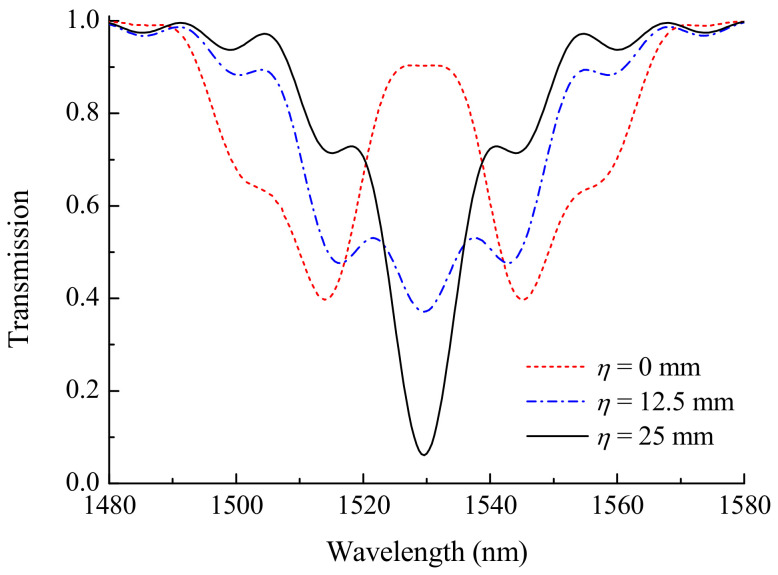
Transmission of grating for LP_05_ cladding mode, when the acoustic front is in its center $z_{0}=L/2$, for η=0, 12.5, and 25 mm ($s_{0}=18$ mstrain).

Figure 4 shows the influence of smoothness parameter η on grating spectrum. The plots are presented for three magnitudes of the parameter η=0, 12.5 to 25 mm for the case of acoustic front in the center of the grating and strain $s_{0}=18$ mstrain. It can be seen that two separate peaks merge in one, when the smoothness becomes comparable with the grating length.

**Figure 5:**
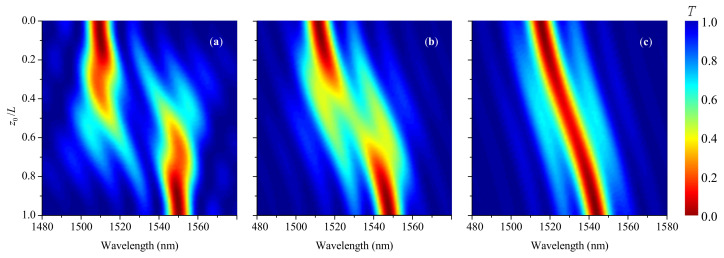
Evolution of transmission spectra with transition point $z_{0}$ moving through the grating for three magnitudes of smoothness η: (**a**) 0, (**b**) 10 mm, and (**c**) 20 mm ($s_{0}=18$ mstrain).

Figure 5 shows evolution of grating spectra with $z_{0}$ moving through the LPFG for three values of smoothness η: 0, 10 mm, and 20 mm ($s_{0}=18$mstrain). The dip transits continuously and smoothly from one wavelength to another, when the smoothness is 20 mm. In this case, interference effects are not observed. For η≈10 mm, the shifting process in wavelength is accompanied by the appearance of a three-prong fork shape and splitting of the spectrum, when the front is in the middle of the grating. For step-changed shock front with η=0, one dip splits into several smaller disappearing dips, and a new dip appears at a shifted wavelength with multiple interference fringes around the main dips.

**Figure 6:**
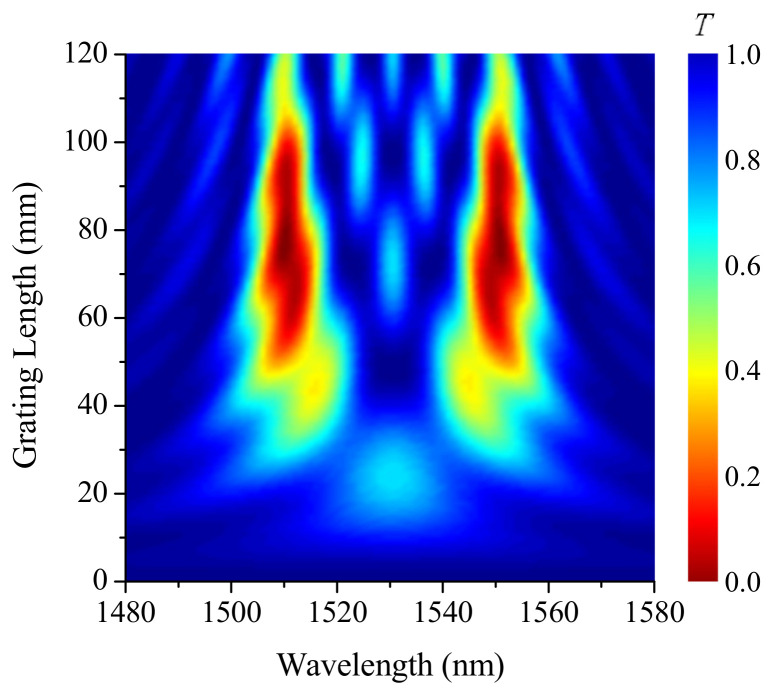
Evolution of transmission spectra with increasing grating length, when the acoustic front is in its center $z_{0}=L/2$, for η=0 mm and $s_{0}=18$ mstrain.

Let us consider the dependence of spectral characteristics of the LPFG with an acoustic front in its center. When the length increases, the transmission loss grows overall. The spectrum is wide for short grating lengths and forms a band centered at 1530 nm (as illustrated in Figure 6). Then, for lengths above 40 mm, two dips are formed at the wavelength corresponding to the strained and non-strained parts of the grating. Further increasing length results in more narrow dips and the appearance of higher sidelobes. This behavior is similar to the behavior of spectra of uniform long-period gratings. Something like a spectral standing wave with increasing number of sidelobes is formed between two main dips.

**Figure 7:**
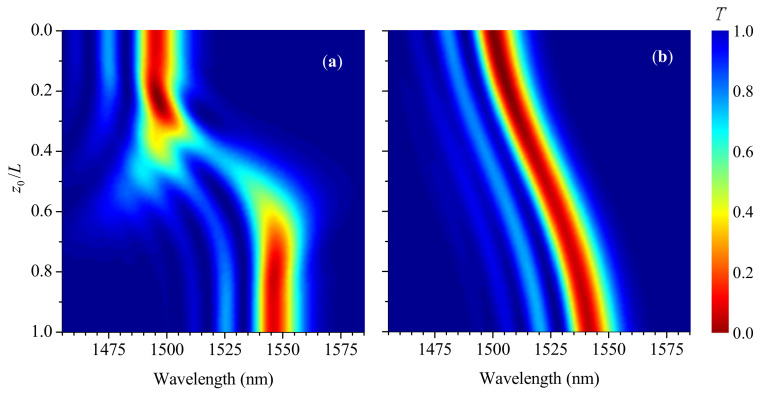
Evolution of transmission spectra for raised-cosine grating and two magnitudes of smoothness (**a**) η=0 and (**b**) 25 mm ($s_{0}=24$ mstrain).

In practice, some apodization can be applied to gratings to produce a smoother spectrum. This is described in our case by the function σ(z). As an example, we calculated the transmission spectrum of the LPFG with raised-cosine apodization (Figure 7). It is similar to the spectrum of the uniform grating but has no spectral sidelobes on long-wavelength side and dipper sidelobes on short-wavelength side. For the stepwise wave front, the transition of the dip from the initial wavelength to the shifted wavelength is asymmetric and irregular, as compared to uniform grating. There is also an effect of loss concentration at $z_{0}/L=0.23$ and wavelength 1497 nm. For η=25 mm, one main dip is formed closer to the center wavelength, and it is accompanied by smaller dips on the short-wavelength side. Compared to Figure 5, no interference fringes are seen on the long-wavelength side of the picture.

## 5. Simulation of π-Shift LPFG

The structure of π-shift LPFG is shown in Figure 1b. We assume that the phase shift is in the center of the grating. For the simulation of transmission, we divide the whole structure into two parts. Then, we obtain solution of the coupled mode equations in the first part, which gives us the amplitudes of the core and cladding modes. Next, we add a phase shift to one mode and solve the coupled mode equations in the second part of the grating. Finally, we find transmission coefficient from the amplitudes of the modes at the end of the grating.

The spectrum of a π-shift LPFG has a transmission maximum in the center of the grating with two rejection bands on the sides, while the maximum is the result of destructive interference is caused by the introduction of the phase shift at the center. The same phase shift produces two rejection bands that are formed by constructive interference at the side wavelengths. The maximum between the two rejection bands is largest when the phase shift is at the center of the grating; if the amplitude of modulation in the grating is the same, the two rejection bands are shallower, compared to the single band the uniform grating.

When the acoustic front propagates through the phase-shifted LPFG, the fiber grating is divided at first into three sections: unstrained before phase shift, unstrained after phase, strained after phase. After the wave passes the phase shift, the three sections are the following: unstrained before phase shift, strained before phase, and strained after phase shift.

**Figure 8:**
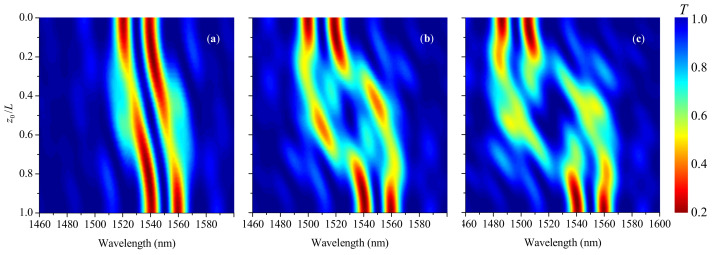
Evolution of transmission spectra with transition point $z_{0}$ moving through the π-shift LPFG for different strains: (**a**) $s_{0}=9$, (**b**) 18, and (**c**) 24 mstrain (η=0).

Evolution of the LPFG’s spectrum with the front moving along the grating is presented in Figure 8. We can see from the figure that the acoustic front shifts the two dips of the π-shift LPFG to the short-wavelength side of the spectrum. For small strain, the shift is accompanied by flow of intensity of one dip into another. With increasing strain, the gap between the dips becomes several times wider than for unstrained grating, while for high strain, the transition of the dips becomes discontinuous; they dissolve at the initial wavelengths and appear at the new wavelengths. The sensitivity of the π-shift LPFGs to the acoustic wave is similar to that of the uniform LPFGs; at the same time, the regular spectrum with a continuous shift of the dips is destroyed at lower strains due to narrower dips.

**Figure 9:**
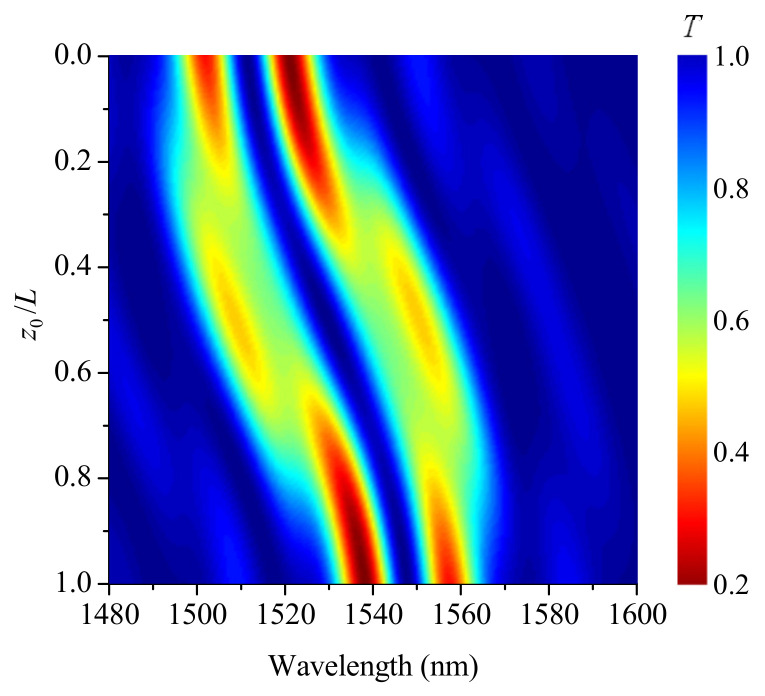
Evolution of transmission spectra with transition point $z_{0}$ moving through the π-shift LPFG for smoothness η=10 mm (strain $s_{0}=18$).

Figure 9 shows how the spectra are changed as a result of smoothing of the acoustic wave front. The smoothness parameter in this simulation is 10 mm. It can be seen that the rejection bands become smoother and wider when compared to the stepwise front when the front covers half of the grating. At the same time, the gap between the bands is almost unchanged and is not widened.

## 6. Simulation of Reflective LPFG

**Figure 10:**
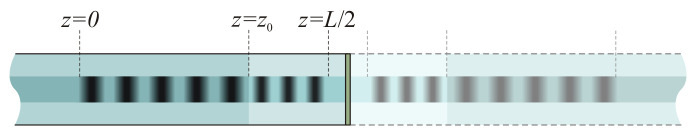
Equivalent scheme of reflective LPFG structure with π-shift.

The reflective LPFG is formed by making a mirror near the end of the grating. Depending on the space between the end of the grating and the mirror, we can obtain the structure that works as either uniform, phase-shifted, or cascaded LPFG. In Figure 10, an equivalent scheme of reflective LPFG is shown with the right side produced by reflecting the left side in the mirror, which is in the center of the figure. Thus, when an acoustic wave propagates through the fiber, the resulting structure represents five sections: non-deformed, deformed, phase shift, deformed, non-deformed. Thus, the simulation was carried out for the LPFG with the account of these five sections. In the equivalent scheme, the acoustic wave propagates from the center to the sides.

**Figure 11:**
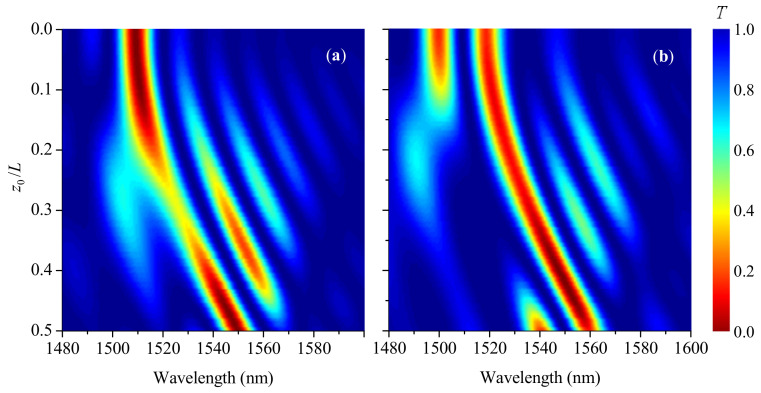
Evolution of transmission spectra with transition point $z_{0}$ moving through the reflective LPFG for strain $s_{0}=18$ with (**a**) zero phase shift and (**b**) π-shift.

When the phase shift is zero, the initial grating is uniform, and its spectrum contains one notch at 1550 nm (Figure 11a); the incoming acoustic wave introduces a phase shift in the middle of the grating and compresses the fiber. As a result, the notch is shifted to shorter wavelengths and split into two when the new notch appears at the initial wavelength, and additional interference dips appear on both sides of the spectrum. When the acoustic front covers the whole grating, the LPFG becomes uniform again and the single notch is reconstituted in a shifted wavelength.

For the reflective π-shift LPFG, the initial spectrum coincides with the spectrum of transmissive π-shift LPFG with two symmetric dips (see Figure 11b). When the acoustic front propagates through the grating, the short-wavelength dip quickly disappears and appears back when the larger part of the grating is strained by the front. The long-wavelength dip shifts almost unaffected. Thus, the reflective π-shift LPFG has a more stable spectral dip that can be used as a signal for sensing applications. In terms of wavelength versus strain sensitivity, different kinds of LPFGS are similar.

## 7. Conclusions

We have studied the spectral behavior of LPFGs deformed by propagating acoustic shock front. Transmission of light through uniform, π-shift, and reflective LPFGs has been simulated using coupled mode theory for co-propagating core and cladding modes with inhomogeneous coupling coefficients. We have shown that, in general, a compressive acoustic shock front propagating through various LPFG structures shifts the resonances of cladding modes to shorter wavelength due to strain in the fiber. However, there are more complex spectral effects, which depend on parameters of the acoustic wave and the grating, such as front amplitude and smoothness, location of the front in the grating, magnitude of the phase shift, and the grating length. The spectrum represents a result of interference of the core and cladding modes interacting differently in different parts of the structure: strained and unstrained, before and after phase shift.

When the smoothness of the acoustic front is compared to the grating length, the notch of a uniform LPFG shifts continuously to shorter wavelengths. Otherwise, when front is steep with smoothness parameter that is significantly less than the grating length, the shifting notch splits into two dips with interference fringes around; indeed, LPFGs with the raised-cosine profile have interference fringes at shorter wavelength side. The spectral behavior of uniform LPFGs strained by a propagating shock front is similar to Bragg gratings, but the spectrum of LPFG cannot be smoothed by using cosine apodization at grating edges due to periodic dependence of transmission on the length of LPFG. The period and length of LPFGs is larger than that of fiber Bragg grating, and characteristics of the structure related to the smoothness of the shock front are changed proportionally.

For the π-shift LPFG having split spectral notch, the gap between the dips becomes several times wider than the gap in the unstrained grating. Increasing smoothness of the acoustic front results in continuous transition of the grating spectra with reduced interference effects. The rejection bands become wider and the gap between the bands is not widened.

The notch of the reflective LPFG without additional phase is shifted to shorter wavelengths with appearance of a new notch at the initial wavelength under the moving acoustic front, while the long-wavelength dip of the reflective π-shift LPFG remains almost unaffected by the acoustic front. The reflective π-shift LPFG has advantages for application in sensors due to its more stable spectral characteristics and one fiber connection needed for the sensor. In terms of sensitivity, different types of LPFG structures have similar parameters.

The results of this investigation can be used for creating LPFG sensors based on the measurement of transmission spectra around one of the cladding mode resonances for detecting shock waves. Furthermore, this analysis is crucial to assess the possibility to implement long-period fiber gratings-based sensors in order to detect leakage of medical gases in health care unities (pressure > 10 MPa), by detection of ultrasound waves (frequency < 100 kHz and 40–60 dB) produced in pipeline systems exhibiting different kinds of anomalies.

## Figures and Tables

**Table 1:** Parameters used in simulation.

Parameter	Designation	Numerical Value
Wavelength	$\lambda_{0}$	1550 nm
Core radius	$r_{\mathrm{co}}$	4.2 μm
Core refractive index	$n_{\mathrm{co}}$	1.4494
Cladding refractive index	$n_{\mathrm{cl}}$	1.4440
Strain-optic coefficient	$p_{a}$	0.22
Grating length	L	40 mm
Grating period	Λ	430 μm
Refractive index modulation	σ	$1.48\cdot 10^{-4}$
Effective refractive indices	$n_{\mathrm{co}}^{\left(\mathrm{eff}\right)}$	1.44649
	$n_{5}^{\left(\mathrm{eff}\right)}$	1.44306
Coupling coefficient	$\kappa_{\mathrm{co}-5}$	$1.6\cdot 10^{-4}$ μm^–1^
Strain correction	$\alpha_{\mathrm{co}-\mathrm{co}}-\alpha {\;}_{\nu -\nu }$	–0.01

**Figure A1:**
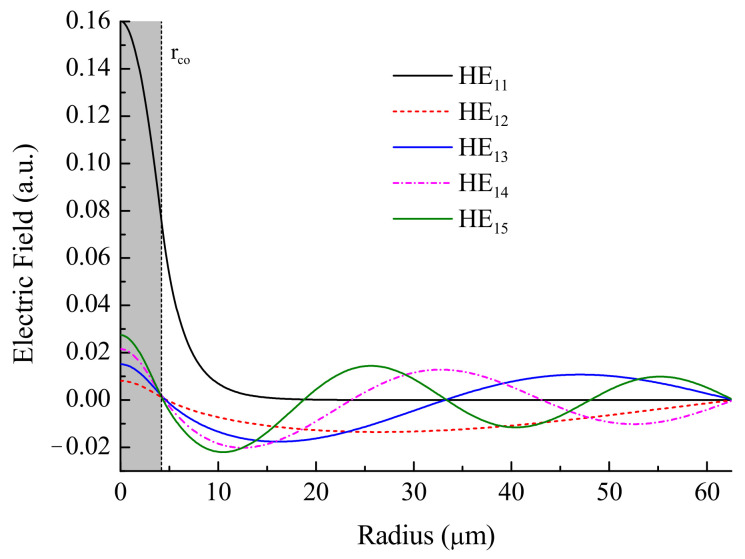
Electric field distributions of five fiber modes. The gray rectangle is the integration area for calculating the overlap integral.

## Data Availability

The processing codes and data segments can be obtained by contacting the corresponding author.

## Appendix A. Calculation of the Cross-Coupling Coefficient

The coupling between the core mode and different cladding modes in the LPFG is determined by the cross-coupling coefficient $\kappa_{\mathrm{co}-\nu }$ (see Equation (16)). We represent this coefficient as function of dimensionless constant $\chi_{\nu }$ expressed through the overlap integral:

(A1) $$\kappa_{\mathrm{co}-\nu }(z)=\chi_{\nu }\;n_{\mathrm{cl}}^{}\sigma (z)k_{0}$$

(A2) $$\chi_{\nu }=\frac{\omega \varepsilon_{0}}{\;n_{\mathrm{cl}}^{}k_{0}}\int_{0}^{r_{\mathrm{cl}}}n_{0}{\left(r\right)}^{2}g(r)E_{\mathrm{co}}^{*}(r)E_{\nu }(r)\pi rdr$$

Assuming that the grating is written in the fiber core, we have g(r)=1 for $r\leq r_{\mathrm{co}}$ and g(r)=0 for $r>r_{\mathrm{co}}$. The refractive index distribution $n_{0}(r)$ in the core is, therefore, equal to $n_{\mathrm{co}}$ and can be approximated as $n_{\mathrm{cl}}$. Then, we obtain

(A3) $$\chi_{\nu }=\frac{\omega \varepsilon_{0}n_{\mathrm{cl}}^{}}{\;k_{0}}\int_{0}^{r_{\mathrm{co}}}E_{\mathrm{co}}^{*}(r)E_{\nu }(r)\pi rdr$$

The electric fields of the modes are normalized to 1 W of power carried by each mode. In this case, the mode fields satisfy the following orthogonality relation:

(A4) $$\int_{0}^{\infty }E_{\mu }^{*}(r)E_{\nu }(r)2\pi rdr=\frac{2\delta_{\mu \nu }\omega \mu_{0}}{\beta_{\nu }}$$

where $\mu_{0}$ is the vacuum permeability, and $\delta_{\mu \nu }$ is the Kronecker delta. In Figure A1, the electric fields of the core mode and four cladding modes are plotted.

By integration of the product of two electric fields over the fiber core, which is depicted a gray rectangle in the figure, we can find the overlap constant $\chi_{\nu }$ for coupling of the core mode with four cladding modes: $\chi_{\nu }=0.030$, 0.055, 0.076, 0.092 for ν=2,3,4,5, respectively. The field amplitude in the core is highest for the core mode HE_11_ and grows with the mode number for the cladding modes HE_1ν_. The overlap constant $\chi_{\nu }$ changes correspondingly.
